# Exophytic botryomycosis: An unusual clinical presentation^☆^

**DOI:** 10.1016/j.abd.2020.10.023

**Published:** 2022-05-21

**Authors:** Lina Paola González-Cardona, Adriana Mercedes Alejo Villamil, Carolina Cortés Correa, Elkin Omar Peñaranda Contreras

**Affiliations:** a Pontificia Universidad Javeriana, Bogotá, Colombia; b Hospital Universitario de la Samaritana, Bogotá, Colombia

Dear Editor,

Botryomycosis is a chronic infectious disease of bacterial origin, granulomatous and suppurative with a worldwide distribution. The incidence and prevalence are unknown, although it is considered an infrequent entity, with approximately 200 cases reported around the world.^1^, ^2^, ^3^

A 42-year-old male patient, a farmer, arrives at the dermatology service with a slow-growing lesion that had appeared 2-years earlier in the great toe finger of the right foot. The patient reported pain of moderate intensity that was enhanced with daily walking, as well as self-limited bleeding. Physical exam reveals in the dorsal aspect of the great toe finger an exophytic ulcerated tumor, erythematous, with hematic crust on the surface and some areas of bleeding, measuring 5×5 centimeters (Fig. 1).

**Figure 1:**
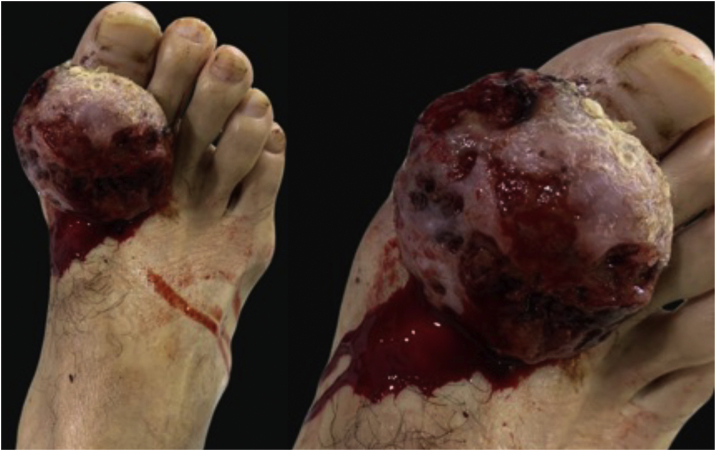
On the right great toe finger exophytic, ulcerated tumor.

A biopsy was taken for the clinical hypothesis of squamous cell carcinoma vs. amelanotic melanoma; the result of the histopathological study showed pseudoepitheliomatous hyperplasia with basophilic granular bodies (grains) with numerous neutrophils (Figure 2, Figure 3). Microbiological cultures were negative. The diagnosis of the exophytic botryomycosis was made, surgical resection was indicated by the plastic surgery service, and antibiotic management with trimethoprim-sulfamethoxazole was started.

**Figure 2:**
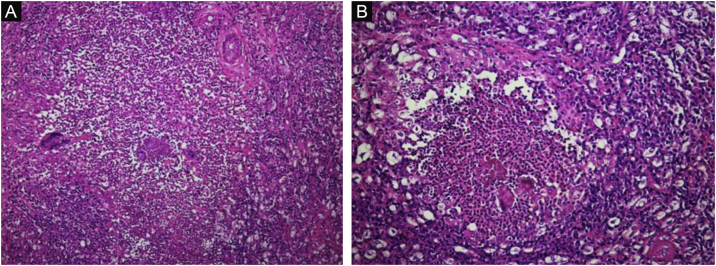
Inflammatory infiltrate with neutrophils and granular basophilic bodies (grains). (A), Hematoxylin & eosin, x10. (B), Hematoxylin & eosin, x40.

**Figure 3:**
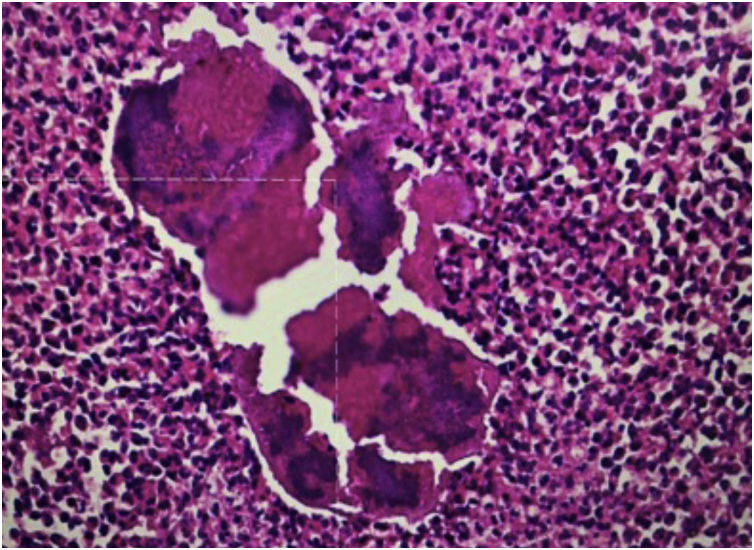
Granular basophilic bodies, grains (Hematoxylin & eosin, x100).

Botryomycosis derives from the Greek “botrys” (bunch of grapes) and “myces” (fungus) because initially, a fungal etiology was suspected. Two types of the presentation can be described, cutaneous and visceral.^1^, ^4^

The cutaneous presentation represents 75% of the reported cases, the remaining 25% correspond to the visceral type. Botryomycosis can occur at any age, although it rarely occurs in ‘children’s and adults over 70-years old, it mainly involves areas with greater susceptibility to trauma such as hands, feet, head and neck.^1^, ^3^, ^4^

The history of trauma is the most important risk factor, in the case presented in this article, this was the probable method of inoculation since the patient works in agriculture. Other risk factors linked are immunosuppression, diabetes *mellitus*, liver disease, alcoholism, systemic lupus, cystic fibrosis, malnutrition, immunoglobulin deficiency, glomerulonephritis, HIV/AIDS, or surgery history.^1^, ^4^

The pathogenesis of this entity is not well understood, and many authors agree that this reaction corresponds to a Splendore-Hoeppli phenomenon, in which antigen-antibody complex, immunoglobulin G and C3 are precipitated, a process in which phagocytosis and intracellular bacterial destruction is prevented.^1^

The 'patient’s present nodules, fistulas, abscesses, and ulcers with seropurulent exudate in which 3‒5 mm white-yellowish granules can be seen and the systemic infection has not been observed.^5^ The diagnosis is made by isolating the causative agent; however, it is not easy to isolate.^1^, ^4^

The differential diagnosis is other infectious granulomatous diseases such as mycetoma, actinomycosis, sporotrichosis, cutaneous tuberculosis, and malignant tumor diseases such as squamous cell carcinoma and amelanotic melanoma.^1^

Antibiotic treatment should be directed to the causative agent, in the case of extensive lesions, failure of systemic treatment, or severely immunocompromised patients, excision and drainage of the lesions are recommended.^1^, ^2^, ^4^ In our case, the treatment was tumor excision by the plastic surgery service with reconstruction with partial-thickness graft, additionally, empirical treatment with trimethoprim-sulfamethoxazole was given due to the epidemiological profile and possible causative agent.

We present the clinical case of exophytic botryomycosis, an unusual clinical presentation previously not reported in the literature.

## Financial support

None declared.

## Authors’ contributions

Lina Paola González-Cardona: Critical literature review; critical manuscript review; preparation and writing of the manuscript; study conception and planning.

Adriana Mercedes Alejo Villamil: Data collection, analysis and interpretation.

Carolina Cortés Correa: Approval of the final version of the manuscript; effective participation in research orientation.

Elkin Omar Peñaranda Contreras: Approval of the final version of the manuscript; intellectual participation in propaedeutic and/or therapeutic; management of studied cases.

## Conflicts of interest

None declared.
